# Orexin system is expressed in avian liver and regulates hepatic lipogenesis via ERK1/2 activation

**DOI:** 10.1038/s41598-020-76329-2

**Published:** 2020-11-05

**Authors:** E. S. Greene, M. Zampiga, F. Sirri, T. Ohkubo, Sami Dridi

**Affiliations:** 1 Center of Excellence for Poultry Science, University of Arkansas, 1260 W. Maple Street, Fayetteville, AR 72701 USA; 2 Dipartimento Di Scienze E Tecnologie Agro-Alimentari, Alma Mater Studiorum-Università Di Bologna, Bologna, Italy; 3 College of Agriculture, Ibaraki University, Ibaraki, 300-0393 Japan

**Keywords:** Molecular biology, Physiology

## Abstract

Orexins are originally characterized as orexigenic hypothalamic neuropeptides in mammals. Subsequent studies found orexin to be expressed and perform pleiotropic functions in multiple tissues in mammals. In avian (non-mammalian) species, however, orexin seemed to not affect feeding behavior and its physiological roles are poorly understood. Here, we provide evidence that orexin and its related receptors are expressed in chicken hepatocytes. Double immunofluorescence staining showed that orexin is localized in the ER, Golgi, and in the lysosomes in LMH cells. Brefeldin A treatment reduced orexin levels in the culture media, but increased it in the cell lysates. Administration of recombinant orexins upregulated the expression of orexin system in the liver of 9-day old chicks, but did not affect feed intake. Recombinant orexins increased fatty acid synthase (FASN) protein levels in chicken liver, activated acetyl-CoA carboxylase (ACCα), and increased FASN, ATP citrate lyase(ACLY), and malic enzyme (ME) protein expression in LMH cells. Blockade ERK1/2 activation by PD98059 attenuated these stimulating effects of orexin on lipogenic factors. Overexpression of ERK1/2 increased the expression of lipogenic genes, and orexin treatment induced the phosphorylated levels of ERK1/2^Thr202/Tyr204^, but not that of p38 ^Thr180/Tyr182^ or JNK1/2 ^Thr183/Tyr185^ in chicken liver and LMH cells. Taken together, this is the first report evidencing that orexin is expressed and secreted from chicken hepatocytes, and that orexin induced hepatic lipogenesis via activation of ERK1/2 signaling pathway.

## Introduction

Orexin A and B^1^ (also known as hypocretins 1 and 2^2^) are 33-amino acid (ORX-A) and 28-amino acid (ORX-B) peptides derived from proteolytic cleavage of a single precursor, prepro-orexin, and were originally discovered to be produced by several feeding-related hypothalamic nuclei in rodents and humans^1,2^. More recently, orexins and their cognate receptors have been found to be expressed in several mammalian peripheral tissues, including the gastrointestinal tract, pancreas, adrenals, kidney, adipose tissue, and the reproductive tract (for review see^3^), suggesting potential pleiotropic functions and involvement in multiple versatile physiological processes (for review see^4^).

The physiological effects of orexin (ORX) are mediated by two G-protein coupled receptors, orexin receptor 1 (ORXR1) and orexin receptor 2 (ORXR2). Originally identified as regulators of feeding behavior^5^, orexins are also known to control sleep–wake cycle^6–8^, energy balance and glucose homeostasis^9,10^, autonomic function^11,12^, and as a modulator of the stress response^13,14^. In adipocytes, orexin has been shown to decrease lipolysis^15^ and increase lipid accumulation^16^, hinting at effects on adipogenesis and lipogenesis.

In avian (non-mammalian) species, although prepro-orexin sequence is highly conserved^17^, and avian orexin system was found in the brain, testis, ovary, stomach, and intestine^18,19^, little is known about its physiological roles. Recently, we have shown the presence of ORX and its receptors, ORXR1 and ORXR2, in the muscle^20^ of avian species and demonstrated its role in modulating energy metabolism and mitochondrial function.

As orexin has been shown to play a key role in lipid metabolism in rodents, and as fat accumulation and liver hemorrhagic syndrome are a source of substantial burden and cost to poultry, we undertook this study to determine the role of orexin in avian lipid metabolism. Since the liver is the primary site for de novo fatty acid synthesis in chicken^21^, we hypothesized that orexin may be produced by the hepatocytes and may regulate lipogenesis. Our data showed that orexin system is expressed in chicken liver and avian hepatocyte cell lines and that orexin administration modulate the expression of lipogenic genes. The effect of orexin on avian hepatic lipogenesis seems to be mediated via extracellular signal-regulated kinase 1/2 (ERK1/2) signaling pathways.

## Materials and methods

### Animals

Day-old male broilers (Cobb 500, Cobb-Vantress, Siloam Springs, AR) were divided into 3 weight-matched groups (39.75 ± 0.26 g), reared for 8 days, and have ad libitum access to clean water and feed (4.090 Mcal ME kg^−1^ and 22% CP). The ambient temperature was reduced gradually (1 °C every 2 days) from 32 to 29 °C, and the relative humidity was maintained at 55 ± 2%. Feed and water intake were recorded hourly. On day 9, birds received an intraperitoneal (i.p.) injection of recombinant human orexin-A or -B (rORX-A or -B; Alpha Diagnostic International, San Antonio, TX, 100 ng/100 g BW, n = 12). The control group received a saline solution. Three hours after administration, birds were euthanized by cervical dislocation. Blood was collected and 250 µL added to Trizol LS reagent (Life Technologies, Carlsbad, CA) for mRNA extraction. Liver samples were immediately collected, snap frozen in liquid nitrogen and stored at − 80 °C for further analysis. The present study was conducted in accordance with the recommendations in the guide for the care and use of laboratory animals of the National Institutes of Health and the protocols were approved by the University of Arkansas Institutional Animal Care and Use Committee under protocol # 16084.

### Leghorn Male Hepatoma (LMH) cell culture and treatments

LMH cells (ATCC CRL-2117) or spontaneously immortalized chicken embryonic hepatocytes (siCEH^22^) were grown on 0.1% gelatin-coated culture dishes in Waymouth’s media (Life Technologies, Carlsbad, CA), supplemented with 10% FBS (Life Technologies, Carlsbad, CA), and 1% penicillin–streptomycin (BioBasic, Amherst, NY) at 37 °C, in a humidified atmosphere of 5% CO_2_ and 95% air. At ~ 80% confluence, cells were synchronized with serum-free media overnight and treated with different doses (0, 10, and 100 nM) of recombinant human ORX-A or ORX-B (Alpha Diagnostic International, San Antonio, TX) for 24 h. The dose of orexins was selected based on pilot and previous studies^16,20,23,24^. 30 min before orexin treatments, LMH cells were incubated with brefeldin A (0.3 µg/mL) for 24 h and lysates and medium were subjected to immunoblot analyses. All culture experiments were performed with cells from passages 10–15 and repeated at least twice.

### Transient overexpression and inhibition of ERK1/2

At 50–60% confluence, LMH cells were pretreated with PD98059 (10 µM, ThermoFisher Scientific, Waltham, MA) 30 min before orexin administration. Cells were transiently transfected with pEGFP-ERK1, pEGFP-ERK2, and pEGFP empty vector (mock) using Lipofectamine 2000 (ThermoFisher Scientific, Waltham, MA) according to the manufacturer’s instructions. Six to eight hours post transfection, complete medium was added and cells were maintained for additional 16 h and were processed for immunoblotting.

### RNA isolation and quantitative real-time PCR

For chicken liver and cell lysates, total RNA was extracted using Trizol reagent (Life Technologies, Carlsbad, CA) according to the manufacturer’s instructions, and concentration and quality were determined by using the Take3 micro-volume plate and the Synergy HT multimode microplate reader (BioTek, Winooski, VT). The ratio of absorbance (A260/280) as well as agarose gel electrophoresis were used to assess RNA integrity and quality. RNA was reverse transcribed using qScript cDNA Sythesis Supermix (Quanta Biosciences, Gaithersburg, MD), and amplified by qPCR (Applied Biosystems 7500 Real Time System) with SYBR green master mix (Life Technologies, Carlsbad, CA) as previously described^25–27^. Relative expression of the target genes was determined using the 2^−ΔΔCT^ method, with normalization to *r18S* expression^28^. Oligonucleotide primers specific for chicken are summarized in Table 1.

**Table 1:** Oligonucleotide qPCR primers.

Gene	Accession number^a^	Primer sequence	Orientation	Product size (bp)
ORX	AB056748	CCAGGAGCACGCTGAGAAG	For	67
		CCCATCTCAGTAAAAGCTCTTTGC	Rev	
ORXR1	NM_001024584.1	TGCGCTACCTCTGGAAGGA	For	58
		GCGATCAGCGCCCATTC	Rev	
ORXR2	XM_004945362	AAGTGCTGAAGCAACCATTGC	For	61
		AAGTGCTGAAGCAACCATTGC	Rev	
ACC	NM_205505.1	CAGGTATCGCATCACTATAGGTAACAA	For	74
		GTGAGCGCAGAATAGAAGGATCA	Rev	
FAS	NM_205155.2	ACTGTGGGCTCCAAATCTTCA	For	70
		CAAGGAGCCATCGTGTAAAGC	Rev	
ME	NM_204303.1	AGATGAAGCTGTCAAAAGGATATG	For	62
		CACGCCCCTTCACTATCGA	Rev	
ACLY	NM_001030540.1	CTTTTAAGGGCATTGTTAGAGCAAT	For	65
		CCTCACCTCGTGCTCTTTCAG	Rev	
SCD-1	NM_204890.1	CAATGCCACCTGGCTAGTGA	For	52
		CGGCCGATTGCCAAAC	Rev	
SREBP-1	NM_204126.2	CATCCATCAACGACAAGATCGT	For	82
		CTCAGGATCGCCGACTTGTT	Rev	
SREBP-2	XM_015289037.2	GCCTCTGATTCGGGATCACA	For	63
		GCTTCCTGGCTCTGAATCAATG	Rev	
PPARα	NM_001001464.1	CAAACCAACCATCCTGACGAT	For	64
		GGAGGTCAGCCATTTTTTGGA	Rev	
PPARγ	NM_001001460.1	CACTGCAGGAACAGAACAAAGAA	For	67
		TCCACAGAGCGAAACTGACATC	Rev	
INSIG1		TGGCGCTGGTGCTGAAC	For	63
		TGACCTCGTCGGGAAACAG	Rev	
INSIG2	NM_001305465.1	CAGCGCTAAAGTGGATTTTGC	For	65
		CAATTGACAGGGCTGCTAACG	Rev	
SCAP	XM_025147369.1	TGGCCCAGAGACTCATCATG	For	67
		GCAGGATCCGTATAAACCAGGAT	Rev	
ERK	NM_204150.1	CGGACCATGATCACACAGGAT	For	63
		CAGGAGCCCTGTACCAACGT	Rev	
JNK	NM_205095.1	GCCGATGATCAGCCAGGAT	For	62
		GGCCCAATGGAAGCAAGAG	Rev	
p38	XM_419263.5	AGCTGGAGATTGAGGAATGGAA	For	62
		CGGTGGCACAAAGCTGATTA	Rev	
18s	AF173612	TCCCCTCCCGTTACTTGGAT	For	60
		GCGCTCGTCGGCATGTA	Rev	

^a^Accession number refer to Genbank (NCBI).

### Conventional PCR

Total RNA was extracted and reverse transcribed as described above. Fragments of *ORX*, *ORXR1/2*, and r*18S* were amplified by PCR using specific primers^20^. PCR was performed using 43 μL of Platinum PCR SuperMix (Life Technologies, Carlsbad, CA), 5 μL of cDNA, and 1μL of each forward and reverse primers. Thermal cycling parameters for *ORX* and *ORXR1/2* were: 94 °C for 4 min, followed by 39 cycles of 94 °C for 30 s, 50 °C for 30 s, 72 °C for 1 min, with a final extension at 72 °C for 10 min. For r*18S*, the temperatures were the same, but the annealing and elongation steps were each held for 1 min. Fragments were separated on a 1% agarose gel electrophoresis and images captured using the FluorChem M MultiFluor System (ProteinSimple, San Jose, CA ).

### Western blot analysis

Liver tissue and cells were homogenized in lysis buffer (10 mM Tris Base, pH 7.4, 150 mM NaCl, 1 mM EDTA, 1 mM EGTA, 0.1% Triton X-100, 0.5% NP-40, protease and phosphatase inhibitors). Protein concentrations were determined using a Bradford assay kit (Bio-Rad, Hercules, CA) and a Synergy HT multimode microplate reader (BioTek, Winooski, VT). Proteins (80 μg liver tissue, 40 μg cell lysate) were run on 4–12% gradient Bis–Tris gels (Life Technologies, Carlsbad, CA), and transferred to PVDF membranes. The membranes were blocked with 5% non-fat milk in TBS-T for 1 h at room temperature, then incubated with primary antibodies (1:500–1:1000 dilution) overnight at 4 °C. Secondary antibodies (1:5000) were diluted in 5% milk in TBS-T and membranes were incubated at room temperature for 1 h. Primary antibodies used were: rabbit anti-ORX, rabbit anti-ORXR1, rabbit anti-ORXR2 (Interchim, France), rabbit anti-phospho-ACCα^Ser79^, rabbit anti-ACCα, rabbit anti-FASN, rabbit anti-phospho-ERK1/2^Thr202/Tyr204^, rabbit anti-ERK1/2, rabbit anti-phospho-SAPK/JNK^Thr183/Tyr185^, rabbit anti-SAPK/JNK, rabbit anti-phospho-P38 MAPK^Thr180/Tyr182^, rabbit anti-P38 MAPK (Cell Signaling Danvers, MA), and rabbit anti-nucleoline (Santa Cruz Biotechnology, Dallas, TX ). To assess protein loading, the expression of the housekeeping GAPDH protein was determined using rabbit anti-GAPDH antibody (Santa Cruz Biotechnology, Dallas, TX). The signal was visualized by chemiluminescence (ECL Plus, GE Healthcare, Pittsburg, PA) and captured by the FluorChem M MultiFluor System (ProteinSimple, San Jose, CA).

### Immunofluorescence

Immunofluorescence was performed as previously described^25^. Briefly, cells were grown in chamber slides and fixed with methanol for 10 min at − 20 °C, then permeabilized with Triton-X 100. Cells were blocked with serum-free protein block (Dako, Carpinteria, CA) for 1 h at room temperature, then incubated with anti-ORX, anti-ORXR1, anti-ORXR2, anti-phospho-ACC^Ser79^ or anti-phospho-ERK1/2^Thr202/Tyr204^ (1:200, in Antibody Diluent, Dako, Carpinteria, CA, overnight at 4 °C) for mono-staining or a combination of anti-ORX with anti-ERGIC53 or anti-TGN38 for double-labeling immunofluorescence. For ER or lysosome co-labeling with ORX, cells were incubated with 100 nM of ER-Tracker or Lyso-Tracker (ThermoFisher Scientific, Waltham, MA) for 15–30 min before fixation and then proceeded as mentioned above. Signal was visualized with DyLight 488-conjugated secondary antibody (Thermo Fisher Scientific, Grand Island, NY). Slides were cover slipped with Vectashield with DAPI (Vector Laboratories, Burlingame, CA), and images were obtained and analyzed using Zeiss Imager M2 and AxioVision software (Carl Zeiss Microscopy).

### Statistical analysis

Data were analyzed by one-way ANOVA. Significant differences among individual group means were determined by Student–Newman–Keul’s multiple range test with GraphPad Prism v. 6.00 (La Jolla, CA). Significance was set at α = 0.05. All data are represented as means ± SEM.

## Results

### Orexin system is expressed in avian liver cells

**Figure 1:**
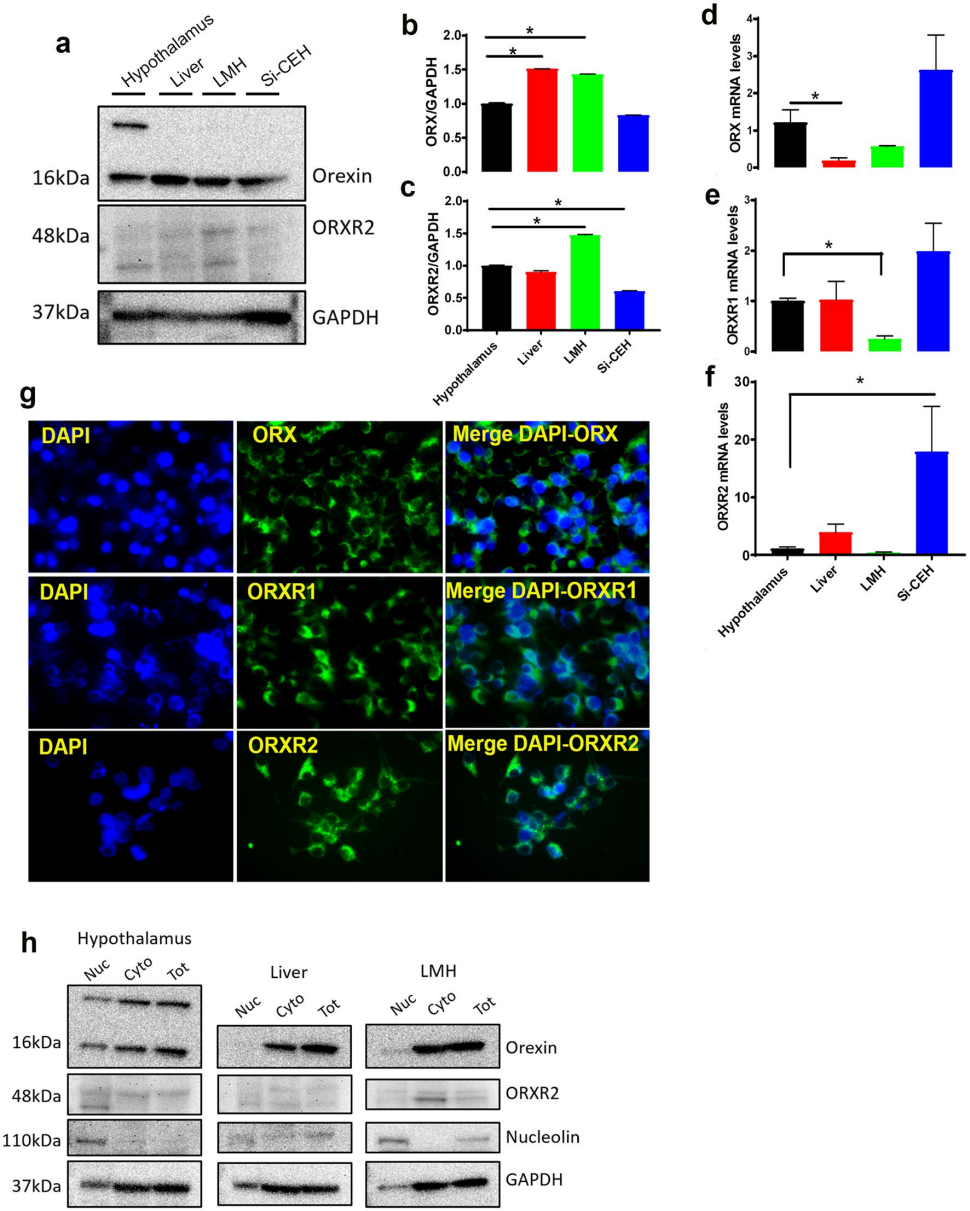
Orexin and its related receptors are expressed in chicken liver and hepatocyte cells. (**a**–**c**) Total proteins were isolated from chicken hypothalamus (positive control), liver, LMH, and siCEH cells and subjected to Western blot using anti-orexin and anti-ORXR2 antibodies and data are expressed as ratios to GAPDH. (**d**–**f**) Total RNA was reverse transcribed and subjected to RT-qPCR as described in the “Materials and methods” with chicken hypothalamus served as a calibrator, and relative expression was determined by 2^−ΔΔCt^ method^28^. (**g**) Distribution of intracellular orexin system was determined by immunofluorescence staining with secondary antibody conjugated with AlexaFluor 488 (green) and DAPI (blue). (**h**) Proteins of cellular components (nucleus, cytoplasmic, and total) were obtained by cell fractionation and submitted to Western blot analysis as described above. Nucleolin was used as a positive control for the nuclear fraction. *Significant difference from the hypothalamus, *P* < 0.05. *Cyto* cytoplasmic, *LMH* leghorn male hepatoma cells, *Nuc* nuclear, *siCEH* spontaneously immortalized chicken embryonic hepatocytes, *Tot* total. Full-length blots/gels are presented in Supplementary Fig. S1.

**Figure 2:**
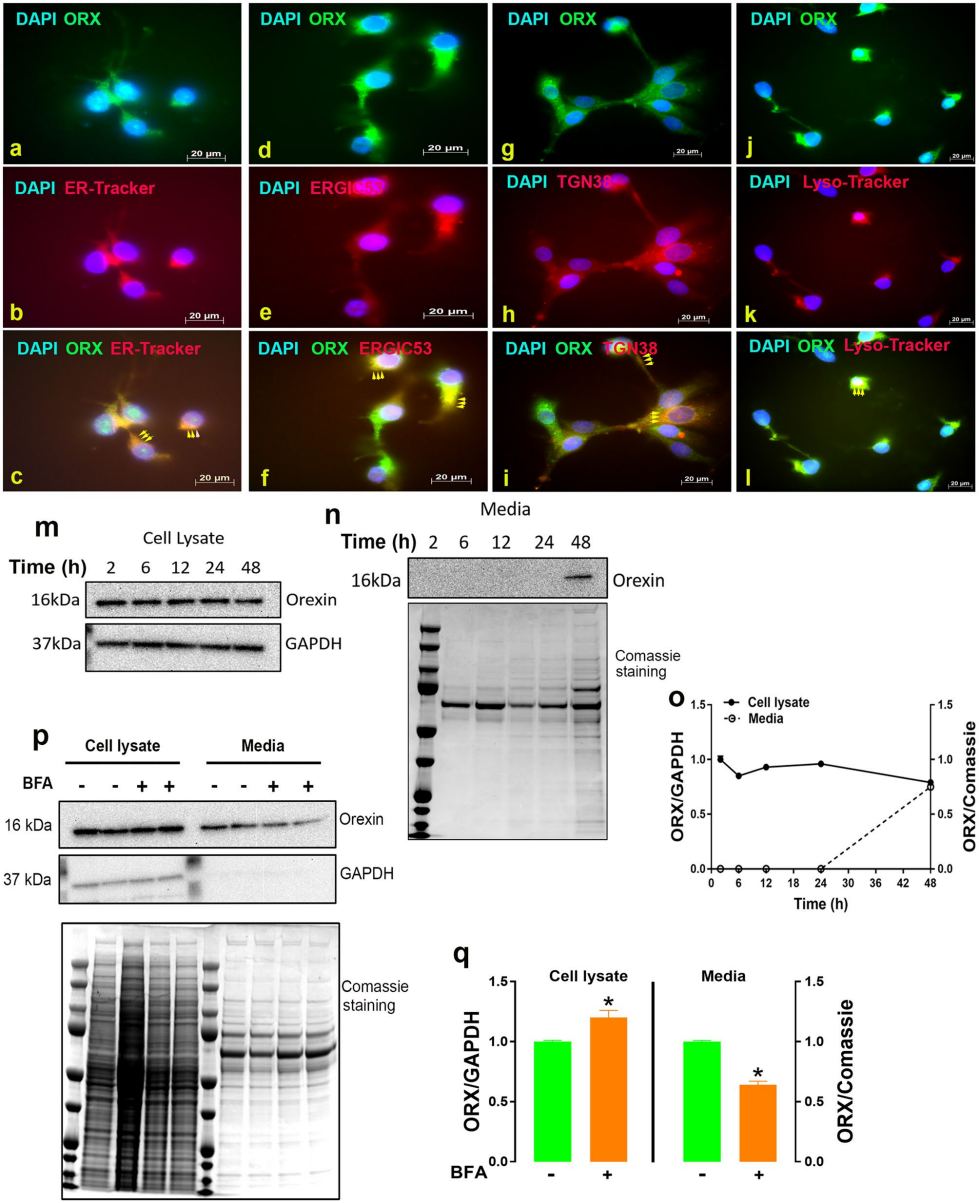
Subcellular localization and secretion of orexin by LMH cells. Chicken orexin was stained in combination with well characterized markers of the ER (ER-Tracker, **a**–**c**), cis (ERGIC53, **d**–**f**) and trans side (TGN38, **g**–**i**) of the Golgi complex, and the lysosome (lyso-Tracker, **j**–**l**). Yellow arrows showed the co-localization of chicken orexin (ORX) with target organelle-markers. Cell monolayers were incubated in serum-free media for indicated time periods, and collected media and cell lysates were subject to Western blot for orexin protein expression (**m**–**o**). Cells were pretreated with brefeldin A and collected media and cell lysates were subjected to Western blot for orexin protein expression (**p**,**q**). Full-length blots/gels are presented in Supplementary Fig. S1.

By using immunoblot with antibody that cross-reacts with chicken orexin system^20^, we amplified a strong signal of orexin with only one band at the predicted size (16 kDa) in chicken liver as well as in LMH and siCEH cells (Fig. 1a). However, we observed two bands with molecular weight of 16 and 230 kDa in the hypothalamus (positive control, Fig. 1a). We amplified also a strong signal of orexin receptors in chicken hepatocytes (Fig. 1a). Orexin protein levels are significantly higher in the chicken liver compared to the hypothalamus (Fig. 1b), however its mRNA abundance are significantly lower (Fig. 1d). The expression (mRNA and protein) of orexin receptor (ORXR2) did not differ between the chicken liver and the hypothalamus (Fig. 1c,e,f). Immunofluorescence staining showed a strong reactivity of ORX and its related receptors (ORXR1 and ORXR2) in the cytoplasm of LMH cells (Fig. 1g). The predominant cytoplasmic localization of chicken orexin system was further confirmed by cell fractionation and Western blot analysis (Fig. 1h). To further determine the sub-cellular localization and distribution of orexin in LMH cells, double-labeling immunofluorescence was performed. Chicken orexin was stained in combination with the well characterized ER fluorescent tracking dye, ER-Tracker, and shows to be predominantly localized in the ER of LMH cell line (yellow arrows, Fig. 2a–c). Immunofluorescence localization of Golgi markers suggests that chicken orexin is also localized, in the cis side (co-staining with ERGIC 53), and in the trans side of Golgi complex (co-staining with TGN38) in LMH cells (yellow arrows, Fig. 2d–i). The co-staining of chicken orexin with the lysosome tracking dye, lyso-Tracker deep red, suggests that chicken orexin is also localized in the lysosome (yellow arrows, Fig. 2j–l). Analysis of conditioned medium by Western blot showed specific secretion of orexin in LMH cells at 48 h (Fig. 2m–o), and treatment of cells with brefeldin A (BFA, 0.3 µg/mL), partially but significantly blocked orexin secretion which is demonstrated by a ~ 26% decrease of ORX levels in the medium and a significant accumulation in cell lysate (Fig. 2p,q).

### Orexin modulates its own expression in chicken liver and LMH cells

**Figure 3:**
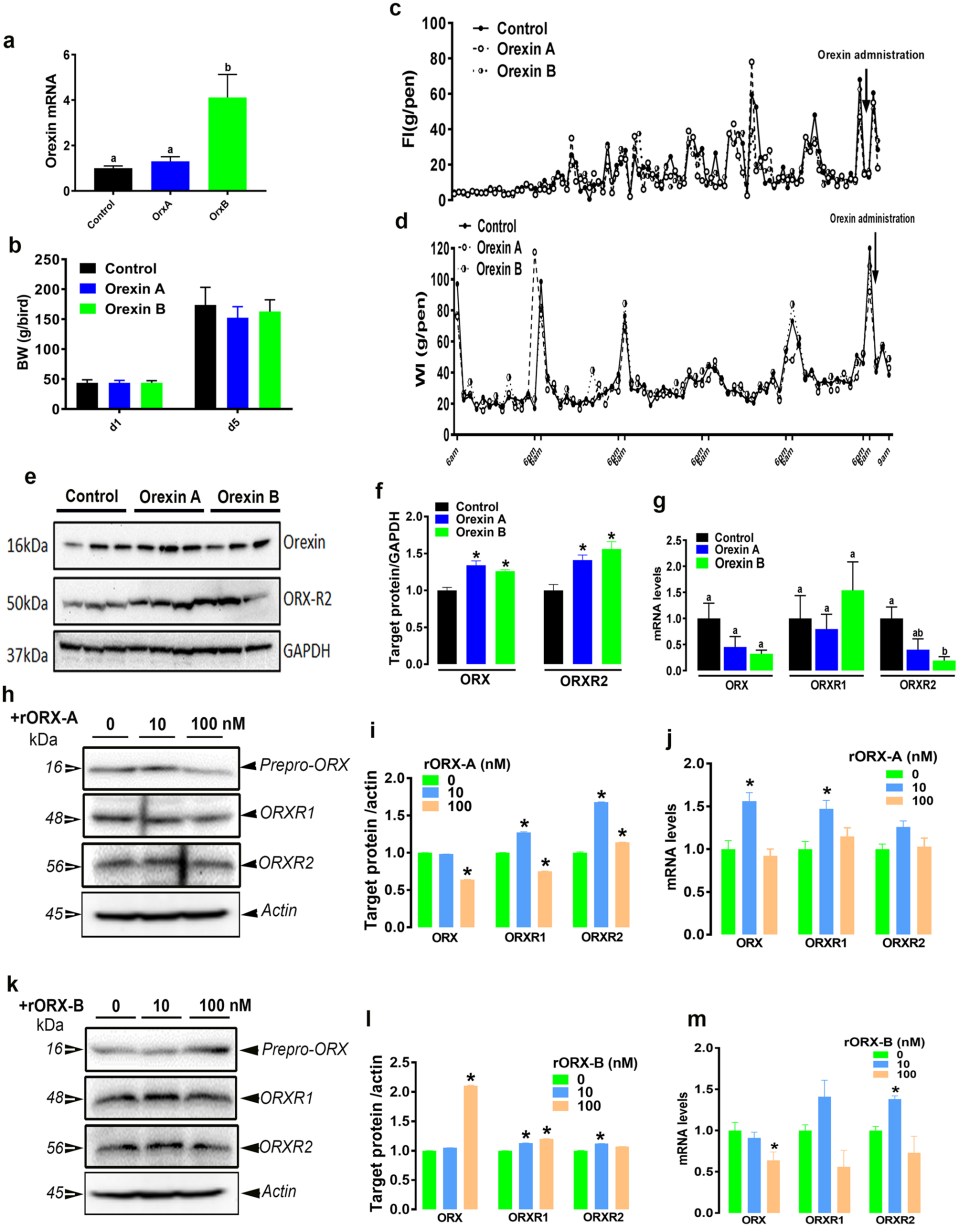
Orexin regulates its own system in chicken liver and LMH cells. Intraperitoneal injection of human recombinant orexins (A and B, 100 ng/100 g BW) affects blood orexin mRNA levels (**a**), BW (**b**), FI (**c**), WI (**d**), and liver expression of orexin and ORXR2 at protein (**e**,**f**) and mRNA levels (**g**). Orexin treatment affects orexin, ORXR1, and ORXR2 at protein (**h**,**i**,**k**,**l**) and mRNA levels (**j**,**m**) in LMH cells. mRNA abundances and protein levels were determined by qPCR and Western blot, respectively as described in “Materials and methods”. Data are presented as mean ± SEM (n = 12 birds/group). *Significant difference from controls, *P* < 0.05. Western blot figures are representative of 4 replicates. *BW* body weight, *FI* feed intake, *WI* water intake. Full-length blots/gels are presented in Supplementary Fig. S1.

Intraperitoneal (IP) administration of recombinant orexin B, but not orexin A, significantly increases ORX mRNA abundances in chick blood (Fig. 3a). Neither recombinant orexin A nor B affect body weight (BW), feed intake (FI), or water consumption (WI) in chicks (Fig. 3b–d). Hepatic ORX and ORXR2 protein levels were significantly increased following administration of recombinant orexins (ORX-A and ORX-B) (Fig. 3e,f). Although, neither hepatic *ORX* nor *ORXR1* mRNA abundances were affected by recombinant orexin treatments, hepatic *ORXR2* gene expression was significantly down regulated by rORX-B administration (Fig. 3g). In LMH cells, treatment for 24 h with 100 nM of rORX-A significantly down regulated prepro-ORX and ORXR1, but increased ORXR2 protein levels (Fig. 3h,i). Low dose of rORX-A increased both ORXR1 and ORXR2 protein expression and upregulated *ORX* and *ORXR1* gene expression in LMH cells (Fig. 3h–j). Treatment with rORX-B at high dose, however, increased ORX and ORXR1 protein levels and down regulated *ORX* mRNA abundance in LMH cells (Fig. 3k–m). Recombinant ORX-B at low dose (10 nM) induced both ORXR1 and ORXR2 protein expression and only *ORXR2* mRNA levels in LMH cells (Fig. 3k–m).

### Orexin regulates lipogenic program in chicken hepatocytes

**Figure 4:**
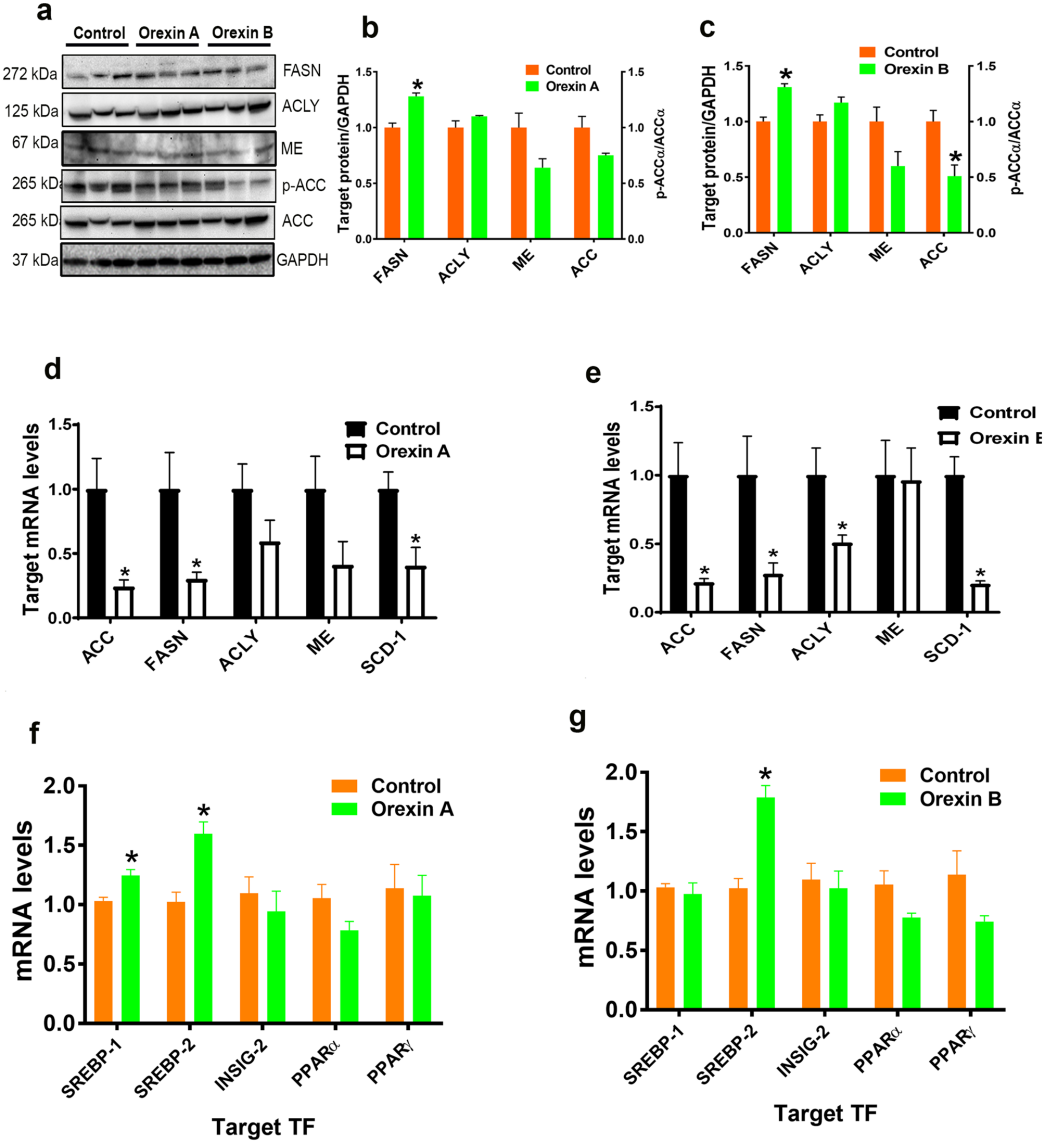
Orexin regulates lipogenic program in chicken liver. Intraperitoneal administration of human recombinant orexins affects lipogenic factors (ACC, FASN, ACLY, ME, SCD-1) and their transcription factors. Protein expression (**a**–**c**) was determined by Western blot and gene expression (**d**–**g**) was measured by qPCR. Data are presented as mean ± SEM (n = 12 birds/group). *Indicates significant difference from controls at *P* < 0.05. *ACC* acetyl-CoA carboxylase, *ACLY* ATP citrate lyase, *FASN* fatty acid synthase, *INSIG-2* insulin-induced gene 2, *ME* malic enzyme, *PPAR* peroxisome proliferator-activated receptor, *SCD-1* stearoyl-CoA desaturase-1, *SREBP-1/2* sterol regulatory element-binding protein 1/2. Full-length blots/gels are presented in Supplementary Fig. S1.

**Figure 5:**
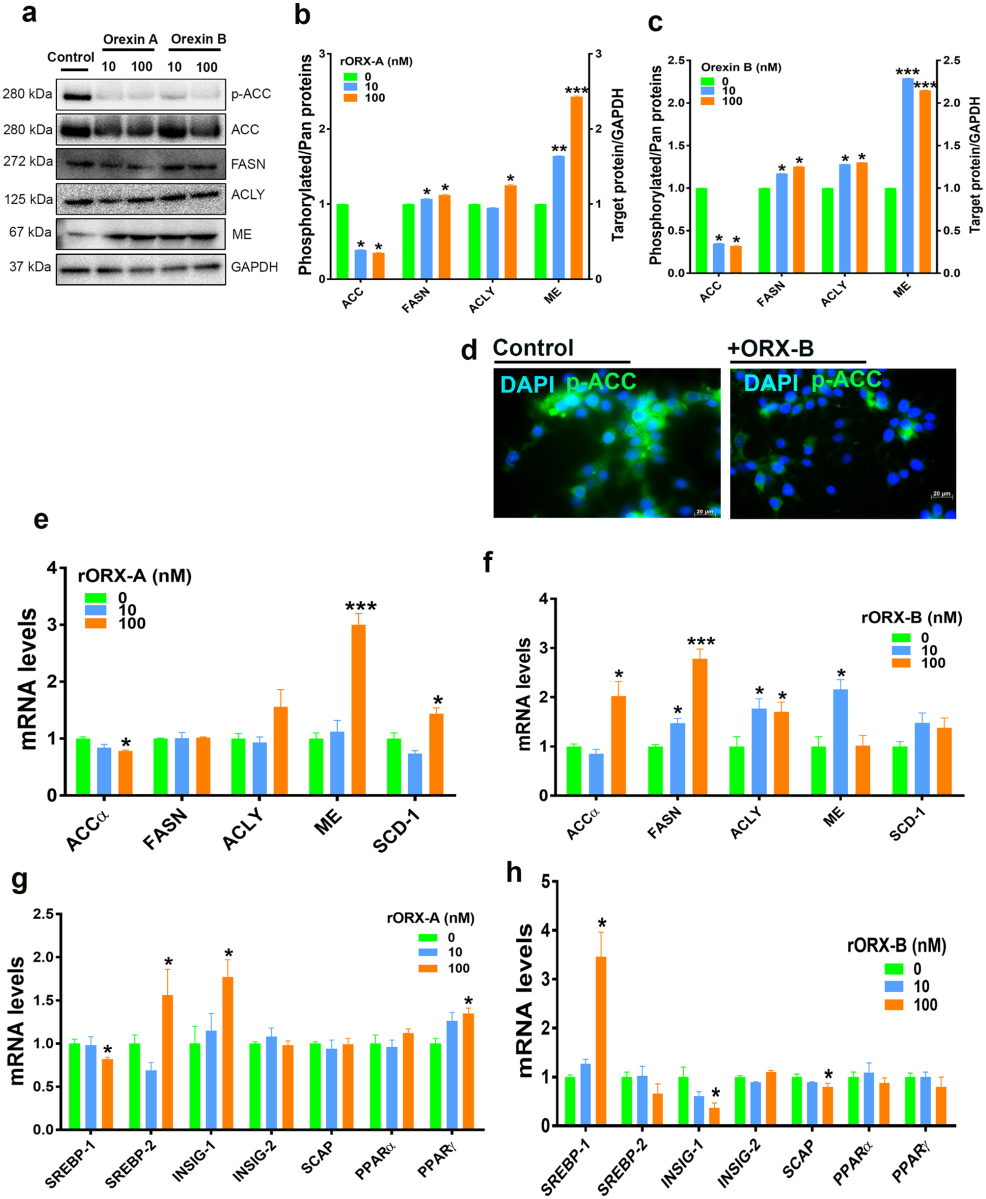
Orexin regulates lipogenic program in LMH cell line. Orexin treatments affects lipogenic factors (ACC, FASN, ACLY, ME, SCD-1) and their transcription factors. Protein expression (**a**–**c**) was determined by Western blot and gene expression (**e**–**h**) was measured by qPCR. Phosphorylated levels of ACC was also assessed by immunofluorescence staining (**d**). Data are presented as mean ± SEM (n = 6/group). *Indicates significant difference from controls at *P* < 0.05. *ACC* acetyl-CoA carboxylase, *ACLY* ATP citrate lyase, *FASN* fatty acid synthase, *INSIG-2* insulin-induced gene 2, *ME* malic enzyme, *PPAR* peroxisome proliferator-activated receptor, *SCD-1* stearoyl-CoA desaturase-1, *SREBP-1/2* sterol regulatory element-binding protein 1/2. Full-length blots/gels are presented in Supplementary Fig. S1.

Administration of recombinant orexin A or B significantly decreased phosphorylated ACCα^Ser79^ and increased FASN protein levels, the rate-limiting enzymes involved in lipogenesis (Fig. 4a–c) and down regulated the expression of *ACCα*, *FASN*, *ACLY*, and *SCD-1* genes in chicken liver (Fig. 4d,e). Recombinant orexin A significantly upregulated the hepatic expression of the transcription factors *SREBP-1* and *SREBP-2* (Fig. 4f), however recombinant orexin B significantly increased only *SREBP-2* mRNA abundances (Fig. 4g). In vitro studies using LMH cells showed that both recombinant orexins at low and high dose significantly reduced the phosphorylated levels of ACCα at Ser79 site (Fig. 5a–c). As shown in Fig. 5d, immunofluorescence staining confirmed the reduced levels of ACCα phosphorylation in LMH cells following recombinant orexin B treatment. Orexin treatments also significantly increased the protein levels of FASN, ACLY, and ME in LMH cells (Fig. 5a–c). At transcriptional levels, recombinant orexin A down regulated *ACCα* and upregulated *ME* and *SCD-1* gene expression in LMH cells (Fig. 5e). Administration of high dose of recombinant orexin B significantly increased the mRNA levels of *ACCα*, *FASN*, *ACLY*, and *ME* (Fig. 5f). These changes were accompanied with a significant down regulation of *SREBP-1* and an upregulation of *SREBP-2*, *INSIG-1*, and *PPARγ* with recombinant orexin A treatment, and an upregulation of *SREBP-1* and a down regulation of *INSIG-1* and *SCAP* with the administration of recombinant orexin B (Fig. 5g, h).

### Orexin B modulates hepatic lipogenesis via ERK1/2 pathway

**Figure 6:**
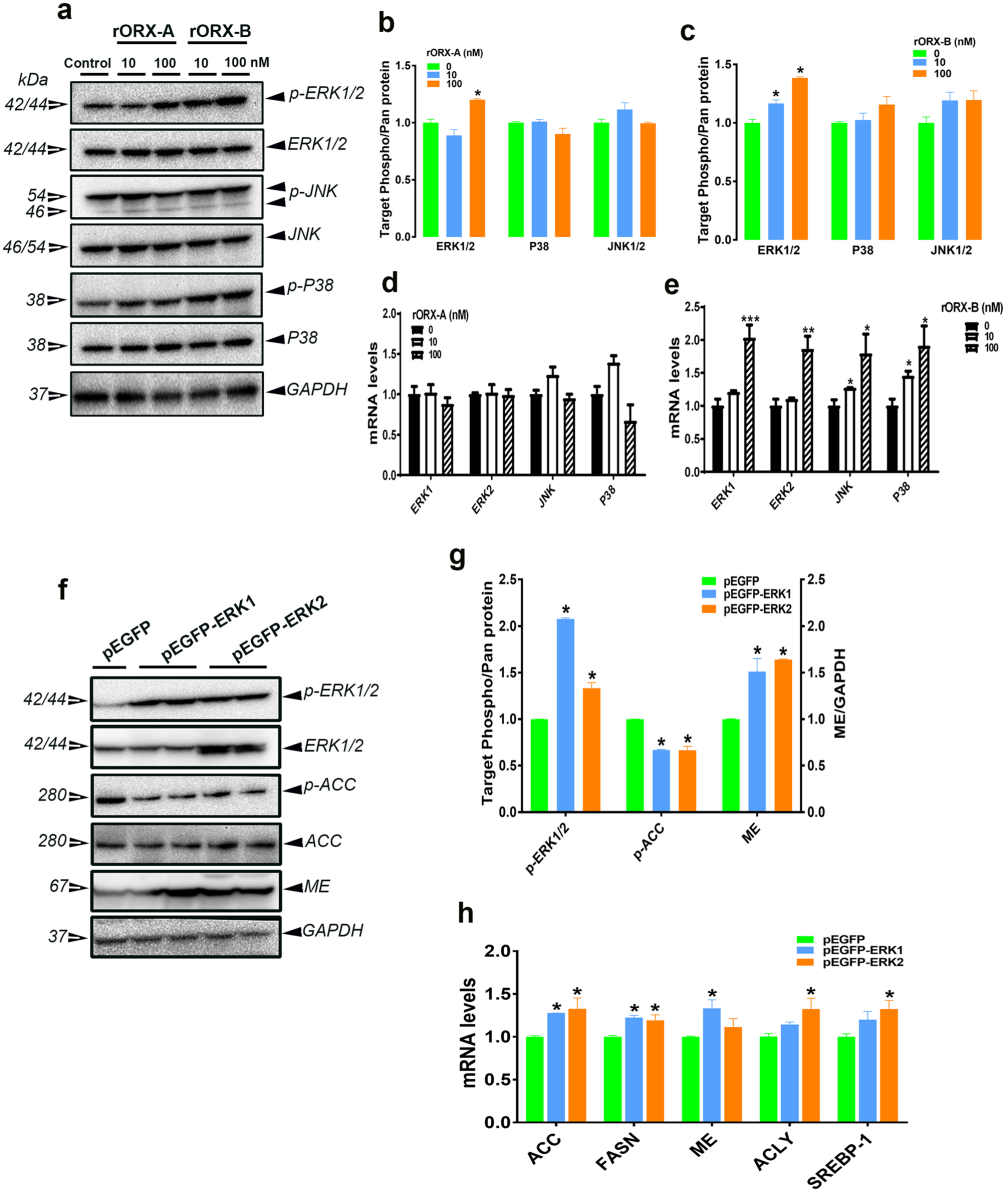
Orexin activates ERK1/2 signaling cascade in LMH cells. Orexin administration increased p-ERK1/2^Thr202/Tyr204^ levels, but not that of p-P38^Thr180/Tyr182^ or p-JNK1/2^Thr183/Tyr185^ in LMH cells as demonstrated by Western blot analyses (**a**–**c**). The relative expression of MAPK genes was measured by qPCR (**d**,**e**). Overexpression of ERK1 or ERK2 increased p-ERK1/2^Thr202/Tyr204^, activated ACC and increased ME protein levels (**f**,**g**) as well as the mRNA abundances of ACC, FASN, ME, ACLY and SREBP-1 (**h**). Data are presented as mean ± SEM (n = 6/group). *Indicates significant difference from controls at *P* < 0.05. *ACC* acetyl-CoA carboxylase, *ACLY* ATP citrate lyase, *ERK* extracellular signal-regulated kinase, *FASN* fatty acid synthase, *JNK* c-Jun N-terminal kinase, *ME* malic enzyme, *P38* P38 mitogen-activated protein kinase, *SREBP-1* sterol regulatory element-binding protein 1. Full-length blots/gels are presented in Supplementary Fig. S1.

**Figure 7:**
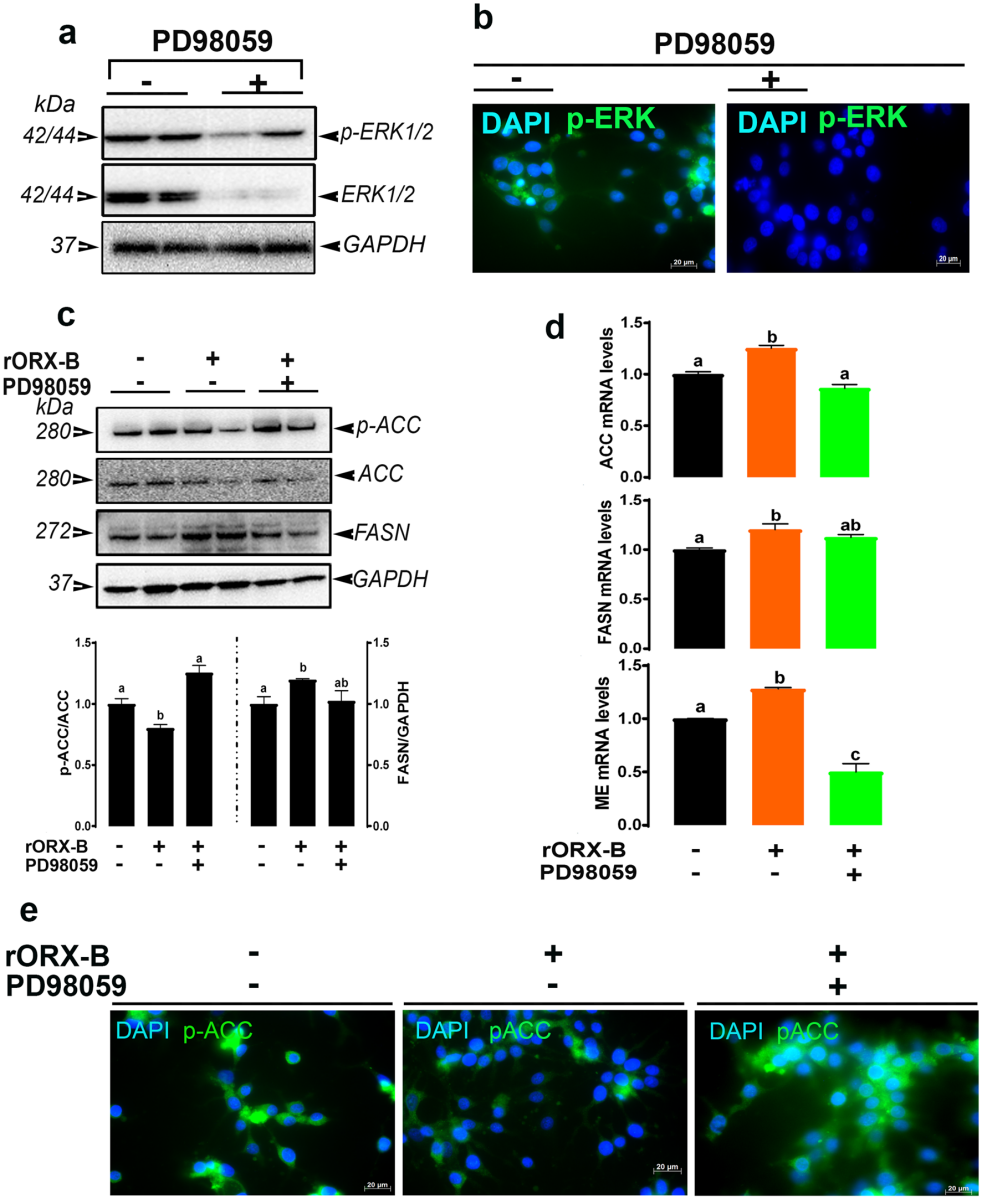
Blockade ERK1/2 activation prevents orexin-induced lipogenesis in LMH cells. Pharmacological inhibition of ERK1/2 using PD98059, as confirmed by Western blot (**a**) and immunofluorescence (**b**), blocked the effect of orexin on the expression lipgenic factors (**c**–**e**). Data are presented as mean ± SEM (n = 6/group). *Indicates significant difference from the untreated group (control) at *P* < 0.05. *ACC* acetyl-CoA carboxylase, *ERK* extracellular signal-regulated kinase, *FASN* fatty acid synthase. Full-length blots/gels are presented in Supplementary Fig. S1.

**Figure 8:**
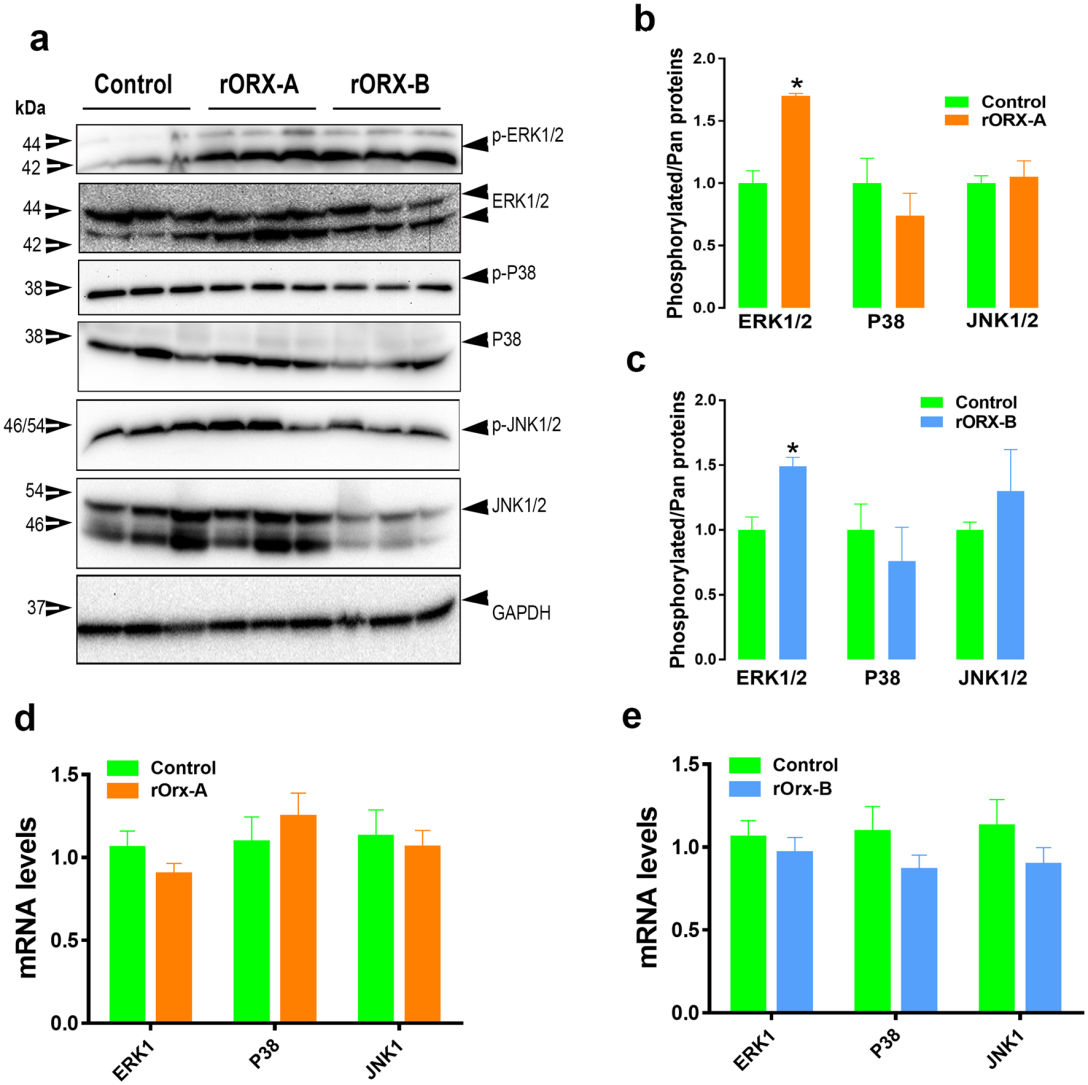
Orexins activate ERK1/2 signaling cascade in chicken liver. Intraperitoneal administration of recombinant orexins increased p-ERK1/2^Thr202/Tyr204^ levels, but not that of p-P38^Thr180/Tyr182^ or p-JNK1/2^Thr183/Tyr185^ in chicken liver as demonstrated by Western blot analyses (**a**–**c**). The relative expression of MAPK genes was measured by qPCR (**d**,**e**). Data are presented as mean ± SEM (n = 12 birds/group). *Indicates significant difference from controls at *P* < 0.05; *ERK* extracellular signal-regulated kinase, *JNK* c-Jun N-terminal kinase, *P38* P38 mitogen-activated. Full-length blots/gels are presented in Supplementary Fig. S1.

Treatment with both doses of recombinant orexin B specifically and significantly induced phospho-ERK1/2^Thr202/Tyr204^ but not that of phospho-p38 ^Thr180/Tyr182^ or phospho-JNK1/2 ^Thr183/Tyr185^ in LMH cells (Fig. 6a–c). High dose of recombinant orexin B significantly up regulated the expression of *ERK1*, *ERK2*, *JNK*, and *P38* genes in LMH cells (Fig. 6e). Administration of recombinant orexin A, however, did not elicit any changes to ERK1/2, p38, or JNK1/2 at both mRNA and protein levels (Fig. 6a,b,d). Overexpression of ERK1 or ERK2 (Fig. 6f,g) significantly decreased phospho-ACCα^Ser79^ and increased ME protein levels in LMH cells (Fig. 6f,g). ERK1 and ERK2 overexpression significantly upregulated the expression of ACCα, FASN, ACLY, and SREBP-1 (Fig. 6h). Pharmacological inhibition of ERK1/2 activation by PD98059 (Fig. 7a,b) blockaded the effects of recombinant orexin B on lipogenic program in LMH cells (increased phospho-ACCα^Ser79^ and decreased FASN protein levels and down regulated *ACCα* and *ME* mRNA abundances) (Fig. 7c–e). Recombinant orexins specifically activated ERK1/2 at Thr202/Tyr204 site, but not P38 or JNK, in chicken liver (Fig. 8a–c). Recombinant orexins did not affect the hepatic expression of *ERK1/2*, *P38*, or *JNK1/2* genes (Fig. 8d,e).

## Discussion

Orexin A and B neuropeptides have attracted much recent attention due to their pleiotropic physiological functions. They were initially characterized in the brain and first emerged as appetite inducers and regulators of feeding behaviors and energy homeostasis in mammals^4^. Numerous studies showed that orexins and their two known G-protein-coupled receptors are expressed in many peripheral tissues and play crucial roles in sleep and wakefulness cycle^6–8^, energy metabolism^9,10^, stress response^13,14^, and glucose and lipid metabolism^23,29^ in mammalian species. Although orexins were found to be expressed not only in the hypothalamus of chickens (non-mammalian species)^17^, but also in gastrointestinal tract^18^, reproductive organs^19^, and muscle^20^, their functions are still not well defined. The objective of the current study was to investigate the potential role of the orexin system in avian hepatic lipid metabolism. As in humans, chicken liver is the primary site of de novo fatty acid synthesis^21^. Additionally, commercial broiler (meat-type) chicken are often hyperphagic and prone to fat accumulation^30^. Since obesity is a worldwide epidemic, these peculiarities make the chicken a high relevant animal model for further understanding hepatic lipid metabolism in both poultry and humans.

By using different techniques (qPCR, immunoblot, and immunofluorescence staining), we first demonstrated that orexin and its related receptors ORXR1 and ORXR2 are expressed in chicken liver and LMH cells. Cell fractionation showed that orexin system is mainly localized in the cytoplasm. Further subcellular analyses by double-labeling immunofluorescence indicated that orexin is localized in ER, Golgi apparatus, and in the lysosome. Although the intracellular distribution of orexin system has not been reported previously, several studies showed that orexin system couples to Ca^2+^ influx and release^31–33^. As the ER plays a key role in calcium homeostasis and storage^34^, together these studies suggested that orexin might be localized in the ER which is supported by COMPARTMENTS, the subcellular localization database (https://compartments.jensenlab.org). Additionally, orexin contains an ER signal sequence^35^. The presence of orexin in ER and Golgi as well as in the culture media indicated that orexin is secreted from chicken hepatocytes. This is confirmed by brefeldin A treatment, the potent inhibitor of secretion via disruption and disassembly of the Golgi leading to the accumulation of orexin in the cell lysate^36^. The localization of orexin in the lysosome suggested that orexin might be involved in autophagosome-lysosome fusion process and autophagy machinery^37,38^.

Although both recombinant orexins upregulated the hepatic orexin system in vivo, rORX-A and rORX-B had differential effects on ORX protein expression in LMH cells. These differences might be due to structural differences between ORX-A and -B (ORX-A contains two disulfide bonds as compared to one in ORX-B) and/or binding affinity of the orexins to their receptors^39^. In fact, mammalian ORXR1 preferentially binds ORX-A, while ORXR2 has similar affinity for both ORX-A and -B^1^. Additionally, heterodimerization with other G-protein coupled receptors is known to affect orexin receptor activation^40^. Determination of the crystal structure of the chicken orexin receptors as well as their ligand-binding properties may help to further elucidate the differential actions of orexin-A and -B^41^. Similarly, changes in mRNA and protein expression of orexin system are not concomitant. For instance, orexin administration upregulated ORX and ORXR2 protein levels without affecting their mRNA abundances. This lack of correlation between mRNA and protein levels is not surprising^42^ and it might be explained by the involvement of varied post-transcriptional and post-translational mechanisms and/or protein and RNA half- lives^43^. The hepatic expression and secretion of orexin system as well as its ability to regulate its own system suggested that orexin might have autocrine, paracrine, and/or endocrine function^44^.

Though originally identified as a regulator of feeding behavior in mammals, orexin does not appear to have this effect in chicken in our study and that of others^45^, leading to questions regarding its role in avian physiology. Here we showed for the first time that orexin administration in vivo increased hepatic FASN protein levels, which is a rate-limiting enzyme of fatty acid synthesis via catalyzing the synthesis of palmitate from acetyl-CoA and malonyl-CoA into long-chain saturated fatty acids^46^. In support of the in vivo data, orexin treatments also induced the expression of key lipogenic proteins including FASN, ACLY and ME in LMH cells. Reduction of its phosphorylated levels indicated the activation of ACCα, which constitutes a key control point in the synthesis of long-chain fatty acids through catalyzing the carboxylation of acetyl-CoA in the generation of malonyl-CoA^47^. In line with our results, previous studies have shown that orexin stimulated lipid accumulation in primary rat adipocytes and 3T3-L1 cells^23^. Pruszynska-Oszmalek and co-workers reported that orexin stimulated proliferation and differentiation of porcine preadipocytes and regulated lipid metabolism^29,48^. It is noteworthy to mention that, as for orexin system and for the same reasons mentioned above, mRNA and protein levels of lipogenic factors were not correlated. Interestingly, in vivo administration of rORX-A up-regulated both hepatic *SREBP-1* and *SREBP-2*, however rORX-B induced only *SREBP-2* gene expression. In LMH cells, however, rORX-A down-regulated *SREBP-1* and up-regulated *SREBP-2* and *INSIG-1* gene expression, and rORX-B increased *SREBP-1* and decreased *INSIG-1* and *SCAP* mRNA abundances. Although the mechanisms contributing to the differential regulation of transcription factors SREBP-1/2 between rORX-A and rORX-B on one hand, and between in vivo and in vitro studies on the other hands, are not known, it is possible that other transcription factors might be involved. For instance, the up-regulation of PPARγ in LMH cells following rORX-A treatment might compensate for the down-regulation of SREBP-1. It has been shown that PPARγ is one of the downstream mediators of orexin’s action^23^. The consistent increase in SREBP-2 mRNA levels following rORX-A administration in both chicken liver and LMH cells indicated that at least rORX-A regulate cholesterol metabolism^49^.

Orexin has been shown to activate various signaling pathways^4,50^ and lipogenic enzymes have been reported to be regulated by diverse mechanisms^51^. In attempt to define the downstream mediators of orexin action on avian hepatic lipogenesis, we sought to determine the involvement of MAPK cascades. Orexin administration led to ERK1/2 phosphorylation at Thr202/Tyr204 residues, but not that of P38 and JNK1/2 in both chicken liver and LMH cells. Overexpression and activation of ERK1 or ERK2 up-regulated the expression of lipogenic genes (ACCα, FASN, ME, and ACLY), and their transcription factor SREBP-1, increased ME protein levels, and activated ACCα as demonstrated by its dephosphorylation. Pharmacological inhibition of ERK1/2 activation by PD98059 prevents orexin-induced lipogenesis. Orexin has been shown to activate ERK1/2 in multiple cell types and species, including CHO cells^52^, SGC-7901 gastric cancer cells^53^, H295R adrenocortical cells^54^, neuro-2a cells^55^, and 3T3-L1 cells^56^. ERK1/2 signaling pathway is involved in a wide variety of cellular processes. Roth and co-workers^57^ have shown that ERK1/2 regulated the key lipogenic transcription factor SREBP-1 and several lines of evidence have implied a link between ERK1/2 and lipid metabolism in mammals^58–61^. Although further in-depth investigations are needed to delineate the mechanisms by which orexin activate ERK1/2 in chicken hepatocytes, we speculate that Gq/Gi, PLC, and PKC are potentially involved^54,62^.

In conclusion, this is the first report evidencing the hepatic expression of orexin system in avian (non-mammalian) species and its role in lipogenesis via ERK1/2 pathway. This is significant because it identified a new molecular signature that could open new vistas for mechanistic understanding of avian hepatic lipid metabolism.

## Supplementary information

Supplementary Information
