# Supplementary material for: Orexin system is expressed in avian liver and regulates hepatic lipogenesis via ERK1/2 activation
Source: Sci Rep. 2020 Nov 5;10:19191. doi: 10.1038/s41598-020-76329-2 (PMC7645691; doi:10.1038/s41598-020-76329-2)

Orexin System is expressed in avian liver and regulates hepatic lipogenesis via ERK1/2 activation

E.S. Greene[1], M. Zampiga[2], F. Sirri[2], T. Ohkubo[3], S. Dridi[1]*

[1]University of Arkansas, Center of Excellence for Poultry Science, Fayetteville, Arkansas 72701

[2]Alma Mater Studiorum – Università di Bologna, Dipartimento di Scienze e Tecnologie Agro-alimentari, Bologna, Italy

[3]College of Agriculture, Ibaraki University, Ibaraki 300-0393, Japan

* Corresponding author: Sami Dridi, Center of Excellence for Poultry Science, University of Arkansas, 1260 W. Maple Street, Fayetteville, AR 72701, USA

Phone: (479)-575-2583, Fax: (479)-575-7139

Email address: dridi@uark.edu

Figure 1

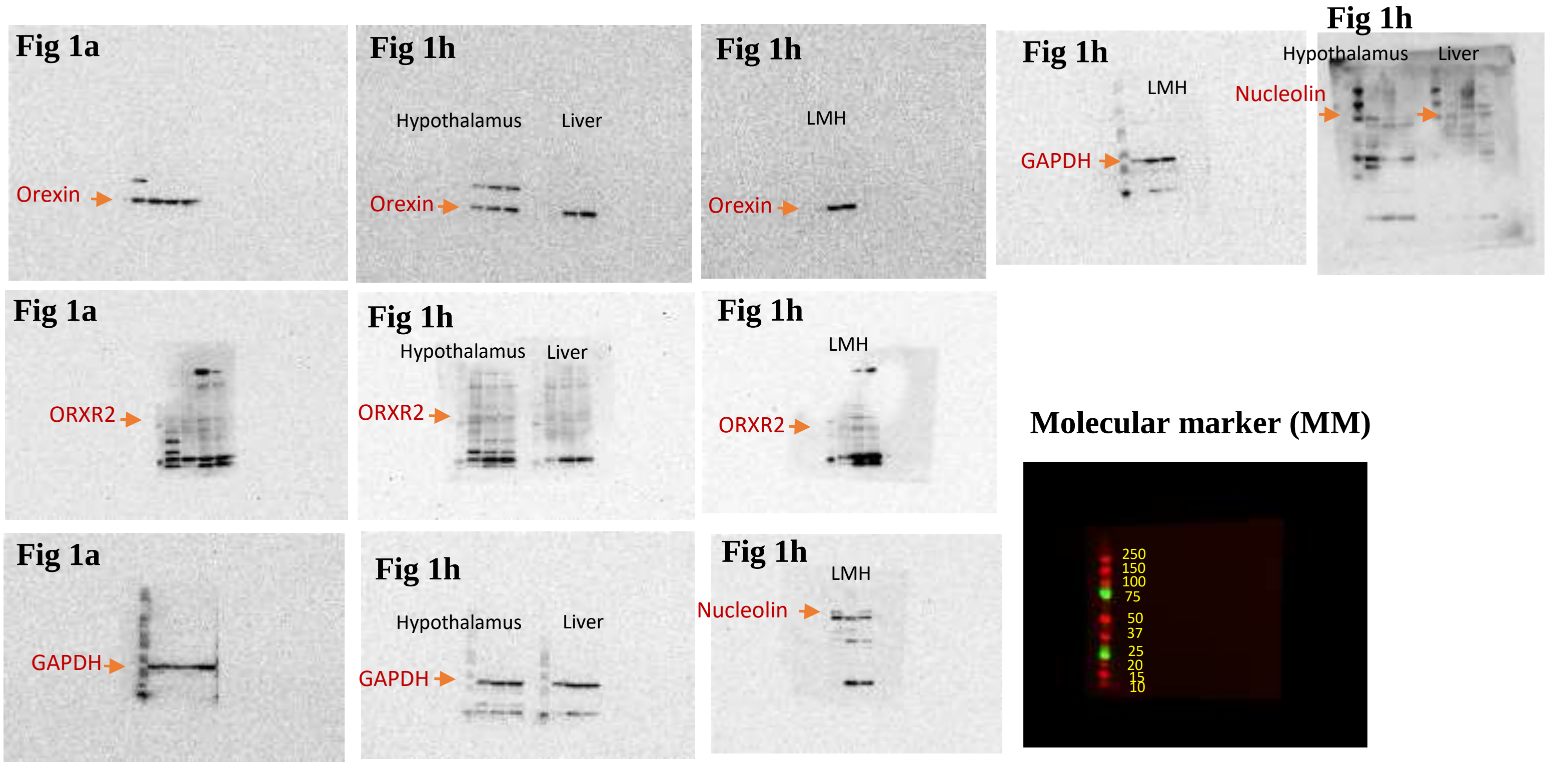

Figure 2

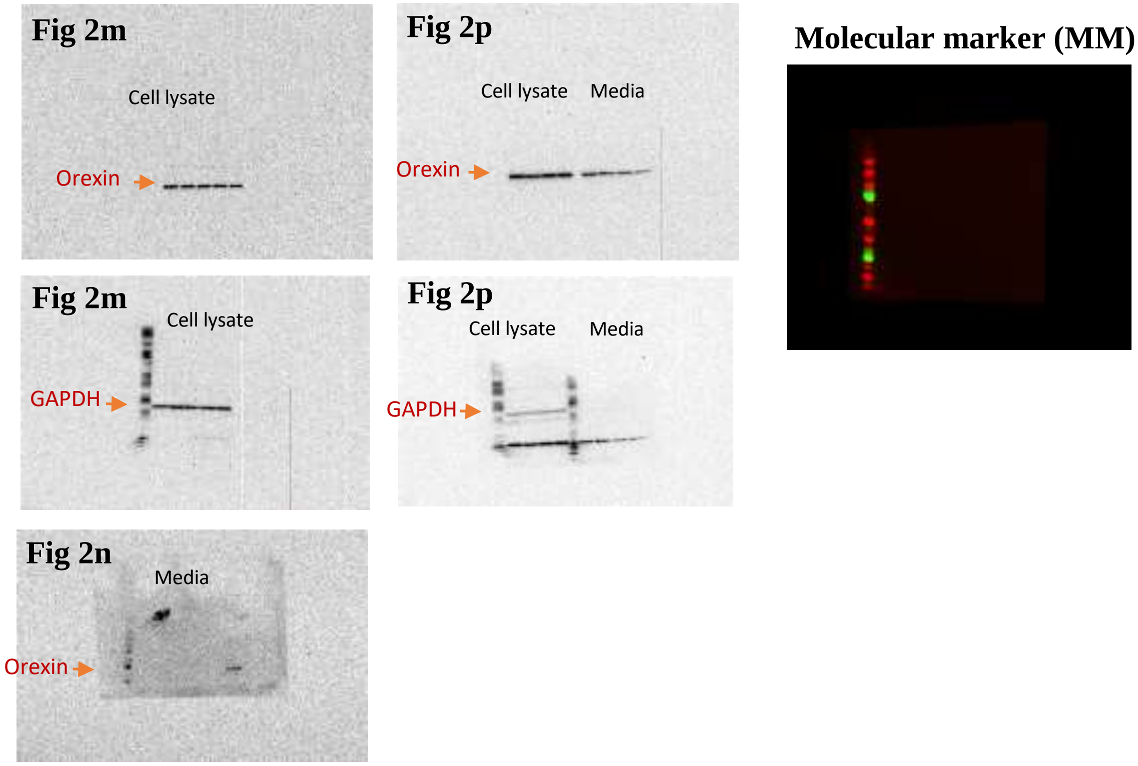

Figure 3

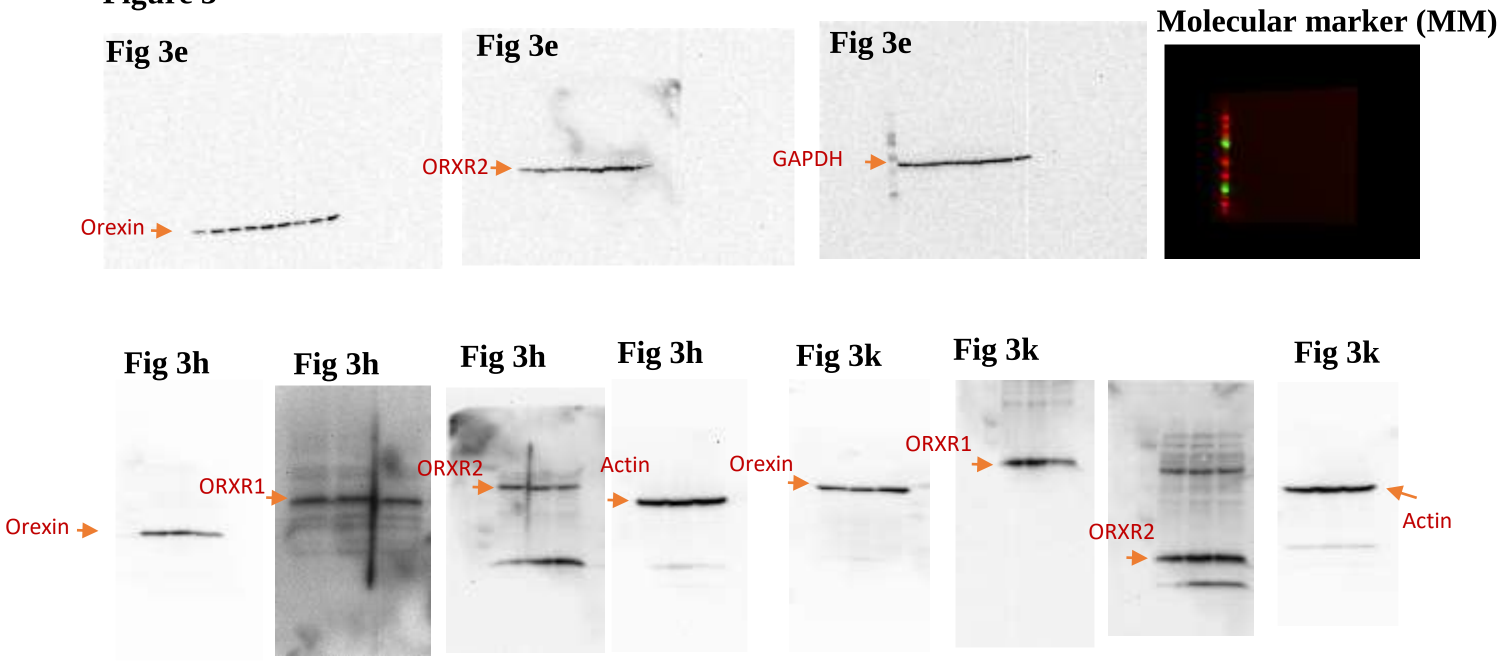
Fig 3e
Orexin
Fig 3e
ORXR2
Fig 3e
GAPDH
Molecular marker (MM)
Fig 3h
Orexin
Fig 3h
ORXR1
Fig 3h
ORXR2
Fig 3h
Actin
Fig 3k
Orexin
Fig 3k
ORXR1
ORXR2
Fig 3k
Actin

Figure 4

Fig 4a

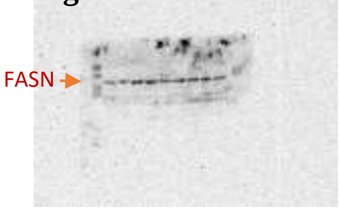

Fig 4a

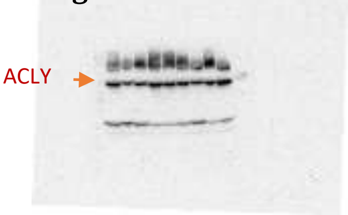

Fig 4a

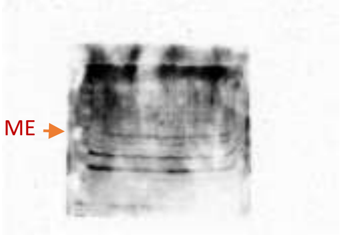

Molecular marker

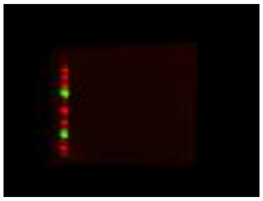

Fig 4a

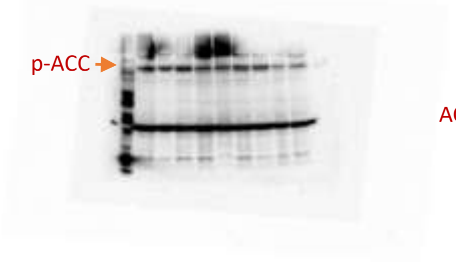

Fig 4a

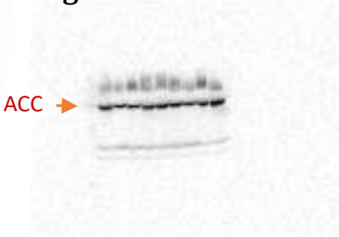

Fig 4a

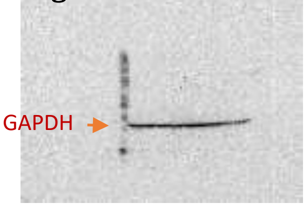

Figure 5

Fig 5a

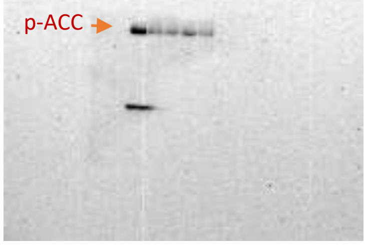

Fig 5a

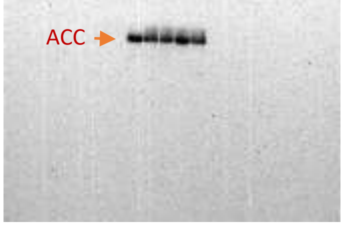

Fig 5a

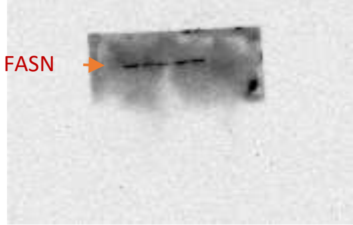

Fig 5a

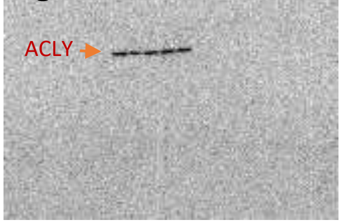

Fig 5a

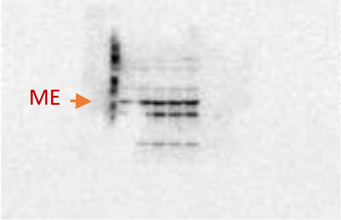

Fig 5a

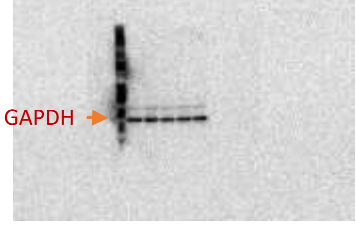

Molecular marker (MM)

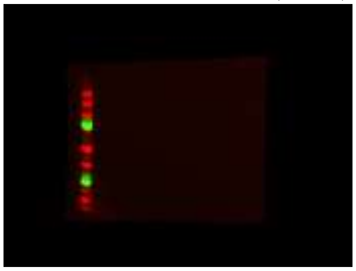

Figure 6

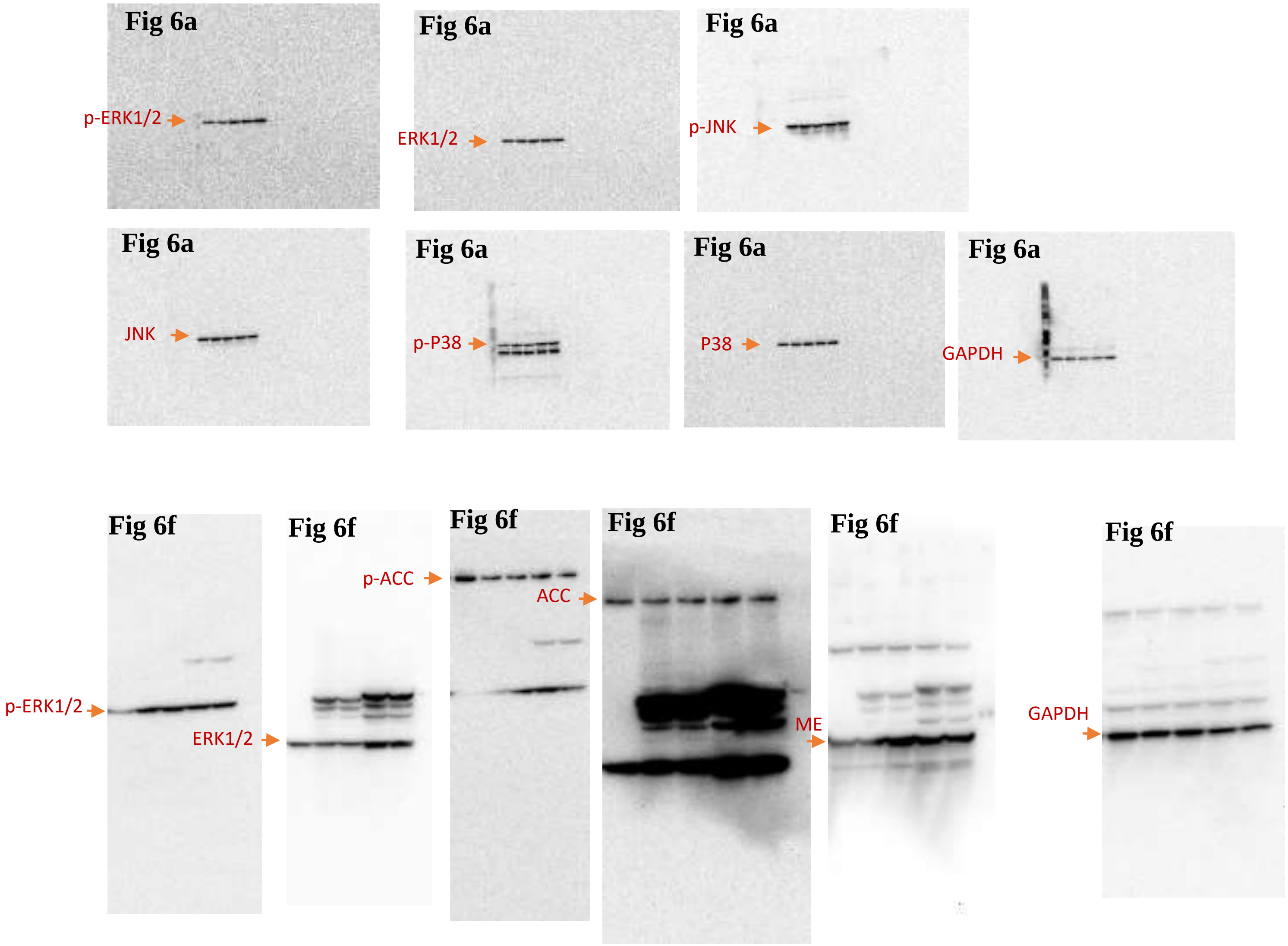

Molecular marker (MM)

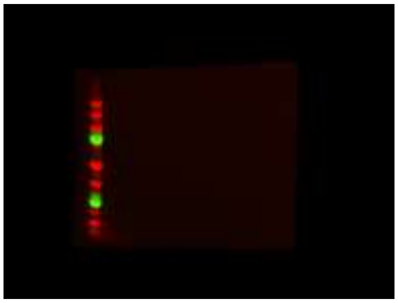

Figure 7

Molecular marker

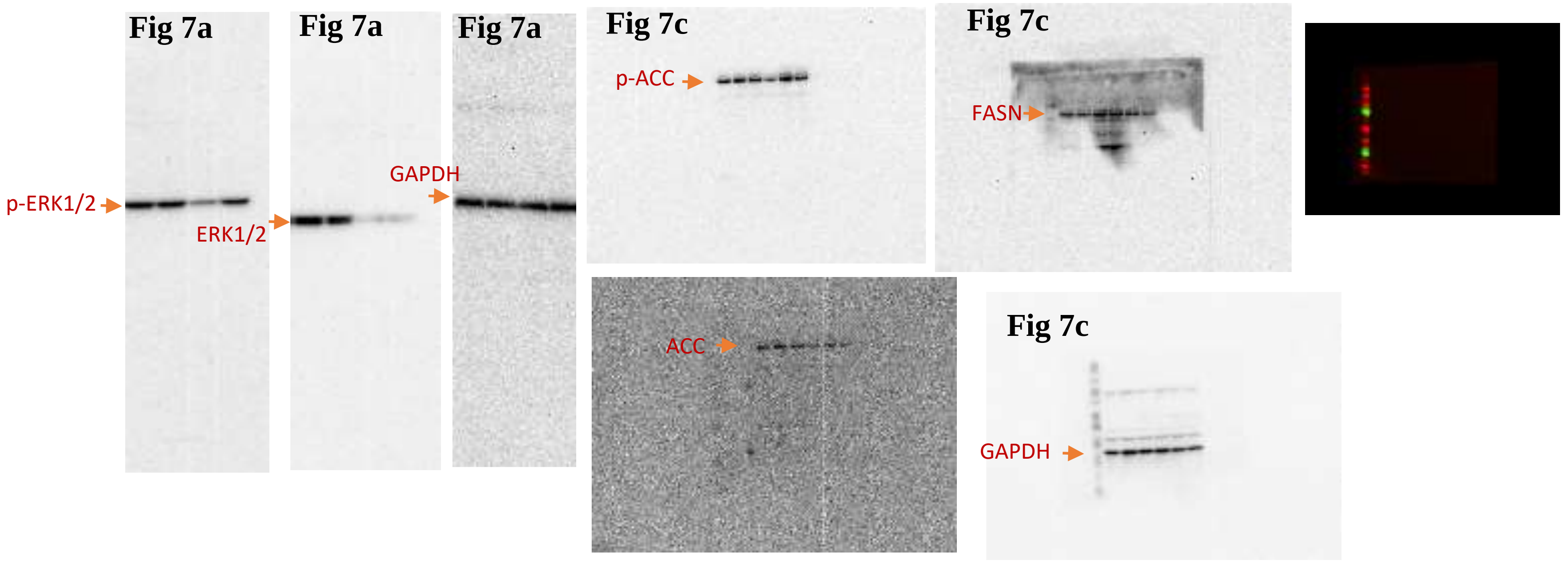

Figure 8

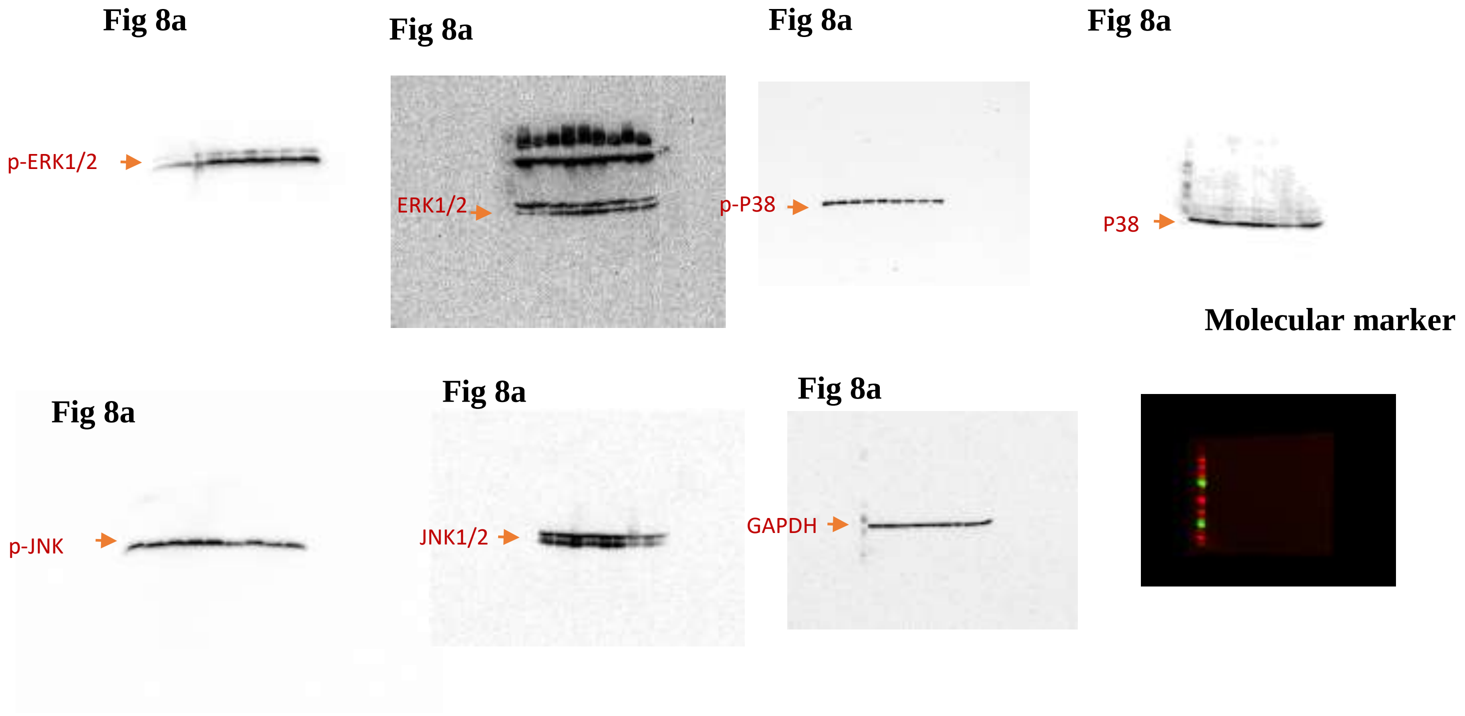

Supplement: Supplementary file 1 — Supplementary Information [file 41598_2020_76329_MOESM1_ESM.pdf]
